# Prediction equation for estimating total daily energy requirements of special operations personnel

**DOI:** 10.1186/s12970-018-0219-x

**Published:** 2018-04-05

**Authors:** N. D. Barringer, S. M. Pasiakos, H. L. McClung, A. P. Crombie, L. M. Margolis

**Affiliations:** 1 Military Nutrition Division, US Army Research Institute of Environmental Medicine, 10 General Greene Avenue, Bldg. 42, Natick, MA 01760 USA; 2 Biophysics and Biomedical Modeling Division, US Army Research Institute of Environmental Medicine, Natick, MA USA; 3 US Army Medical Department Center & School US Army Health Readiness Center of Excellence, US Military-Baylor University Graduate Program in Nutrition, San Antonio, TX USA; 4 Oak Ridge Institute for Science and Education, Oak Ridge, TN USA

**Keywords:** Military, Energy expenditure, Energy balance, Energy deficit

## Abstract

**Background:**

Special Operations Forces (SOF) engage in a variety of military tasks with many producing high energy expenditures, leading to undesired energy deficits and loss of body mass. Therefore, the ability to accurately estimate daily energy requirements would be useful for accurate logistical planning.

**Purpose:**

Generate a predictive equation estimating energy requirements of SOF.

**Methods:**

Retrospective analysis of data collected from SOF personnel engaged in 12 different SOF training scenarios. Energy expenditure and total body water were determined using the doubly-labeled water technique. Physical activity level was determined as daily energy expenditure divided by resting metabolic rate. Physical activity level was broken into quartiles (0 = mission prep, 1 = common warrior tasks, 2 = battle drills, 3 = specialized intense activity) to generate a physical activity factor (PAF). Regression analysis was used to construct two predictive equations (Model A; body mass and PAF, Model B; fat-free mass and PAF) estimating daily energy expenditures.

**Results:**

Average measured energy expenditure during SOF training was 4468 (range: 3700 to 6300) Kcal·d-^1^. Regression analysis revealed that physical activity level (*r* = 0.91; *P* < 0.05) and body mass (*r* = 0.28; *P* < 0.05; Model A), or fat-free mass (FFM; *r* = 0.32; *P* < 0.05; Model B) were the factors that most highly predicted energy expenditures. Predictive equations coupling PAF with body mass (Model A) and FFM (Model B), were correlated (*r* = 0.74 and *r* = 0.76, respectively) and did not differ [mean ± SEM: Model A; 4463 ± 65 Kcal·d^− 1^, Model B; 4462 ± 61 Kcal·d^− 1^] from DLW measured energy expenditures.

**Conclusion:**

By quantifying and grouping SOF training exercises into activity factors, SOF energy requirements can be predicted with reasonable accuracy and these equations used by dietetic/logistical personnel to plan appropriate feeding regimens to meet SOF nutritional requirements across their mission profile.

## Background

Special Operation Forces (SOF), are an elite subset of the military population that regularly engage in prolonged strenuous field operations, resulting in high daily physical activity levels [1, 2] and daily energy expenditures that typically exceed that of the average Service member [3, 4]. Achievement of energy balance is compromised during field operations as energy intake is often confined to short dispersed time periods, and the availability of food is limited to what can be carried [5]. With sustained periods of elevated physical activity and limited food supply it becomes increasingly difficult to consume an adequate amount of energy to match energy expenditure, leading to a net negative energy balance (energy expenditure > energy intake) [6]. This negative energy balance will result in reductions in body mass, and more importantly fat-free mass [7, 8]. Ultimately, if these periods of negative energy balance are of sufficient duration, there will be heightened risk of lower body peak power loss and strength decrements [9, 10], potentially compromising Service members’ ability to optimally perform military tasks and subsequently may impact mission success. Understanding daily energy expenditure is thus a critical factor in developing feeding regimens to minimize the severity of negative energy balance under operational conditions to reduce body mass and fat-free mass losses in an attempt to mitigate declines in physical performance [11].

The capability to accurately predict energy requirements during various SOF training operations is an important first step in developing adequate field feeding programs aimed at minimizing negative energy balance. Given that physical activity level is the main variable dictating daily energy expenditure in SOF personnel [1, 2], it is unlikely that energy expenditure equations developed for use by civilian populations, which are based primarily on body mass, would be accurate for this population [12, 13]. Over the past decade the energy expenditures elicited by a wide range of US SOF training operations have been captured [1, 2, 14]. Compiling data from these multiple studies are thus a source to create an algorithm to predict energy cost of SOF training missions.

The objective of this study is to integrate the raw data collected during 12 SOF training operations into a single dataset to develop an algorithm capable of predicting the energy requirements of personnel engaged in SOF training operations. Additionally, this analysis provides the opportunity to examine the occurrence and severity of energy deficits during SOF training. The outcomes from this analysis will provide useful information to develop appropriate feeding regimens aimed at minimizing negative energy balance during SOF operations.

## Methods

### Participants

Participation in all studies was voluntary, with informed, written consent obtained from each Service member before the initiation of data collection. These studies were conducted after review and approval by the US Army Research Institute of Environmental Medicine Institutional Review Board (Natick, MA). Participant characteristics are presented in Table 1.

**Table 1:** Participant characteristics

Training	*N*	Body Mass	Fat-Free Mass	Fat Mass
Combat Dive School	11	81.6 (76.9, 86.2)	63.8 (60.2, 67.3)	17.8 (15.2, 20.4)
Pre-Mission Training	13	83.1 (79.6, 86.6)	66.1 (63.3, 68.9)	17.0 (14.0, 20.0)
Weapons Training^a^	12	87.6 (82.8, 92.4)	69.3 (64.1, 74.5)	18.3 (14.5, 22.1)
Urban Combat^a^	9	80.8 (75.3, 86.4)	62.1 (57.7, 66.5)	18.7 (15.5, 22.0)
Squad Raids^a^	12	79.67 (75.3, 84.0)	67.9 (63.0, 72.8)	11.8 (9.0, 14.6)
Platoon Raids^a^	11	82.2 (74.0, 90.4)	71.3 (63.0, 79.7)	9.3 (5.4, 13.3)
Small Unit Ranger Training	13	79.6 (75.0, 84.3)	66.2 (63.0, 69.4)	13.4 (10.7, 16.2)
Ranger Selection Assessment Program	17	76.4 (71.8, 81.0)	63.1 (59.1, 67.0)	13.3 (11.9, 14.8)
Raider Spirit^b^	13	84.0 (80.2, 87.8)	70.5 (67.9, 73.1)	13.5 (11.7, 15.3)
Close Quarters Battle^b^	9	87.9 (79.6, 96.2)	71.5 (63.9, 79.0)	16.5 (14.6, 18.4)
Derna Bridge^b^	13	87.3 (83.5, 91.0)	72.4 (69.4, 75.5)	14.8 (12.0, 17.6)
Overall	133	82.2 (80.8, 83.6)	67.4 (66.2, 68.6)	14.8 (14.0, 15.6)

Data presented as mean (95% Confidence Interval of the mean)

^a^Data collected during US Army Special Forces Small Unit Tactics training

^b^Data collected during Marine Special Operations Command training

### Training description

This study integrated data from 12 different SOF training operations. Four trainings were conducted in 2010 at Camp MacKall, NC, as part of the US Army Special Forces Small Unit Tactics training course, a six-week component of the Special Forces Qualification Course [1]. Three trainings took place in 2012 at Fort Benning, GA during the Ranger Assessment and Selection Program (RASP) [14] and Small Unit Ranger Tactics (SURT) training. Three trainings were conducted at Camp Lejeune, NC in 2016 during US Marine Corps Forces Special Operations Command (MARSOC) Individual Training Course (ITC) [15]. One training was performed in 2012 at Special Forces Underwater Operations School in Key West, FL Data, and one at Fort Bliss, TX in 2013 [2]. Data collection during these training operations were generally chosen around major training events, though multiple military tasks were performed during training. Descriptions of training can be found in Table 2.

**Table 2:** Activity characteristics

Training	Description
Combat Dive School	Participants engaged in daily physical training, that included formation runs up to five miles, rigorous callisthenic-type workout, 4 h of high-intensity pool work, open water swims, and drills to properly don 30 kg open circuit diving gear.
Pre-Mission Training	General training of skills required to conduct SOF combat operations; physical training, weapons familiarization, airborne operations, urban operations, and convoy operations
Weapons Training^a^	Training with weapon systems covering the function, mechanics, and employment of the weapon system to include firing the weapon system at firing range
Urban Combat^a^	Military maneuvers in urban terrain comprised mostly of man-made construction such as cities and towns.
Squad Raids^a^	A unit comprised typically of ~ 12 personnel who conduct a rapid attack on an objective followed by a quick movement out of the objective by the attacking force before the enemy can launch a counter attack.
Platoon Raids^a^	A unit comprised typically of ~ 40 personnel who conduct a rapid attack on an objective followed by a quick movement out of the objective by the attacking force before the enemy can launch a counter attack.
Small Unit Ranger Training	Initial fitness tests that include a 5 mile run, push-ups, sit-ups, and a 12 mile ruck march. Classroom taught troop leading procedures, tactics, patrolling techniques, and small unit operations. Field training included a squad sized elements performing mock patrols for multiple days.
Ranger Selection Assessment Program	Initial fitness tests that include a 5 mile run, push-ups, sit-ups, and a 12 mile ruck march. The course is a mixture of classroom, cognitive, and physical assessments to include a field exercise.
Raider Spirit^b^	Simulated raids and ambushes in field settings.
Close Quarters Battle^b^	Weapon qualification and close quarter shoot house training at firing range
Derna Bridge^b^	Training a local guerrilla force and simulated irregular warfare operations in field settings

^a^Data collected during US Army Special Forces Small Unit Tactics Training

^b^Data collected during Marine Special Operations Command

### Anthropometrics and body composition

Vertical height was measured to the nearest 0.1 cm using a stadiometer (Seca; Creative Health Products, Plymouth, MI, USA). Semi-nude (underwear or t-shirt and shorts only) body mass was measured using a calibrated digital scale to the nearest 0.1 kg. Body composition was determined using total body water estimated from doubly labeled water as described below [16].

$$ \mathrm{Fat}\hbox{-} \mathrm{free}\ \mathrm{mass}\ \left(\mathrm{FFM}\right)=\mathrm{total}\ \mathrm{body}\ \mathrm{water}/0.73 $$

$$ \mathrm{Fat}\ \mathrm{mass}\ \left(\mathrm{FM}\right)=\mathrm{body}\ \mathrm{mass}\hbox{--} \mathrm{fat}\hbox{-} \mathrm{free}\ \mathrm{mass} $$

### Energy balance

Energy expenditure for all studies was determined using doubly labeled water (DLW). Total body water (TBW) was calculated using isotopic enrichments from pre to post-dose urine samples [17]

$$ \mathrm{TBW}=\left(\mathrm{A}/{\mathrm{MW}}_{\mathrm{s}}\right)\left({\mathrm{APE}}_{\mathrm{d}}/100\right)\ \mathrm{x}\ 18.02\ \mathrm{x}\ \left[1/{\mathrm{R}}_{\mathrm{s}\mathrm{td}}\left({\mathrm{E}}_{\mathrm{s}}\hbox{--} {\mathrm{E}}_{\mathrm{p}}\right)\right]\ \mathrm{x}\ \left(1/1.01\right) $$

where A is the dose in grams, MW_d_ is the molecular weight of dose water, APE_d_ is the atom percent excess in enrichment of dose water, Rst_d_ is the ratio of heavy to light isotope of standard mean ocean water, and E_s_ and E_p_ are the per milliliter (%) enrichment of the final and pre-dose sample.

All enrichments of ^2^H and ^18^O were measured using isotope ratio mass spectroscopy (Finnigan Mat 252, Thermo Fisher Scientific, Waltham, MA, USA) at Pennington Biomedical Research Center (Baton Rouge, LA). The ^2^H and ^18^O isotope elimination rates (kH and kO) were calculated by linear regression using the isotopic disappearance rates in the urine samples collected over the course of the studies to determine CO_2_ production according to Schoeller et al. [18]:

$$ {\mathrm{rCO}}_2\left(\mathrm{moL}\cdotp {\mathrm{day}}^{\hbox{-} 1}\right)=\left(\mathrm{N}/2.078\right)\ \left(1.01\ \mathrm{kO}\hbox{--} 1.04\ \mathrm{kH}\right)\hbox{--} 0.0246\ \mathrm{rH}2\mathrm{Of} $$

where N is total body water; kO and kH are ^18^O and ^2^H isotope disappearance rates, respectively; and rH2Of is the rate of fractionated evaporated water loss and is estimated to be 1.05 N × (1.01 kO − 1.04 kH). Energy expenditure was calculated using the energy equivalent of CO_2_ for a respiratory quotient of 0.86 based on average food quotient for the course [19].

Resting metabolic rate (RMR) was estimated using measures of fat-free mass (FFM) with the following equation [13]:

$$ \mathrm{RMR}\ \left(\mathrm{Kcal}\cdotp {\mathrm{d}}^{\hbox{-} 1}\right)=370+\left(21.6\ \mathrm{x}\ \mathrm{FFM}\right) $$

Diet-induced energy expenditure was calculated as 10% energy expenditure [20]. Activity-induced energy expenditure was derived from total daily energy expenditure minus RMR and diet-induced energy expenditure [21]. Physical activity level (PAL) was defined as a ratio between energy expenditure and calculated RMR [22].

During SOF training, participants either consumed meals in the dining facility or were provided individual combat rations (meals ready-to-eat (MRE)) or other prescribed food component to consume. No other food or drink outside of what was provided was allowed during the observation periods. When participants ate in the dining facility daily energy intake was determined using the visual estimation technique (Weapons Training, Urban Combat, Squad Raids, and Platoon Raids) [1], digital photography (Ranger Assessment and Selection Program) [14], or dietary recalls (Raider Spirit, Close Quarters Battle, and Derna Bridge) [15]. During field training operations, participants received 3 MREs per day, providing ~ 3900 Kcal·d^− 1^. During RASP participating Soldiers were provided a 600 Kcal dietary supplement and 3 MREs per day, providing ~ 4500 Kcal·d^− 1^. Participants were provided with food logs that contained a list of all the items for each provided ration. Before training, participants were trained to record the percent of each item consumed using the provided logs. Food logs, wrappers and food waste/leftover food from MREs were collected daily to determine consumption. No energy intake data were collected during Combat Dive School and Pre-Mission Training.

### Statistical analysis

Common descriptive statistics were used to characterize body mass and composition, daily energy expenditure, resting metabolic rate, diet-induced energy expenditure, activity-induced energy expenditure, and physical activity levels. Pearson’s correlation coefficients were used to analyze the relationships between daily energy expenditure, energy balance, fat-free mass, fat mass, body composition, and physical activity level. Based on initial analysis, exploratory statistical analysis was used to separate the derived PALs into four quartiles (< 25th percentile, 25-50th percentile, 50-75th percentile, and > 75th percentile) to generate physical activity factors (PAF). The PAF assigned was based on the average physical activity level of each course. The quartiles were low (PAF = 0; mission prep), low-moderate (PAF = 1; common warrior tasks), moderate-high (PAF = 2; battle drills), and high (PAF = 3; specialized intense activities Table 3**)**. Daily energy expenditure predictive equations were constructed using regression analysis, using the constant and β of body mass and physical activity factor for Model A, and constant and β of FFM, and PAF for Model B. Univariate ANOVA with Bonferroni correction was performed to determine if there were any differences between measured and predicted energy expenditures by course. Data were analyzed using IBM SPSS Statistics for Windows (version 22.0; IBM Corp., Armonk, NY). Significance was set at *P* < 0.05, and data are presented as mean (95% confidence interval).

**Table 3:** Physical activity level quartiles

Physical activity level (Training Equivalent)	Training	Percent Daily Energy Expenditure due to Activity^a^
0-Low (< 2.10)	Weapons Training^a^	< 42%
(Briefings/Mission Prep)	Small Unit Ranger Training (classroom)	
	Derna Bridge^b^	
1-Low-Moderate (2.11–2.40)	Pre-Mission Training	42–51%
(Common Warrior Tasks)	Platoon Raids^a^	
	Close Quarters Battle^b^	
2-Moderate-High (2.41–2.75)	Combat Dive School	52–57%
	Squad Raids^a^	
(Battle Drills)	Small Unit Ranger Training (field training)	
	Ranger Selection Assessment Program	
3-High (> 2.75)	Urban Combat^a^	> 58%
(Specialized Intense Activity)	Raider Spirit^b^	

Determined as (activity-induced energy expenditure / daily energy expenditure) × 100

^a^Data collected during US Army Special Forces Small Unit Tactics Training

^b^Data collected during Marine Special Operations Command

## Results

### Energy balance

Mean energy expenditure was 4468 (4311, 4624) Kcal·d^− 1^ for the 12 SOF trainings (Table 4). Physical activity level was associated to daily energy expenditure (*r* = 0.91, *r*^*2*^ = 0.83; *P* < 0.05). Measured energy expenditure was correlated with body mass (*r* = 0.28, *r*^*2*^ = 0.08; *P* < 0.05) and fat-free mass (*r* = 0.32, *r*^*2*^ = 0.10; *P* < 0.05), but not fat mass (*r* = − 0.12, *r*^*2*^ = 0.00; *P* = 0.89). Overall, SOF training resulted in an energy deficit of 28 (23, 33) % daily energy needs equaling a 1433 (1188, 1677) Kcal·d^− 1^ negative energy balance, resulting in a 1.75 (1.40, 2.09) kg decline in body mass (Table 4). Energy balance (*r* = − 0.83, *r*^*2*^ = − 0.69; *P* < 0.05) and change in body mass (*r* = − 0.47, *r*^*2*^ = − 0.21; *P* < 0.05) were both inversely correlated with physical activity level.

**Table 4:** Measured energy expenditure, intake, and balance

Training	Measured Daily Energy Expenditure (Kcal·d^− 1^)	Resting Metabolic Rate (Kcal·d^− 1^)	Diet-Induced Energy Expenditure (Kcal·d^− 1^)	Activity-Induced Energy Expenditure (Kcal·d^− 1^)	Physical Activity Level	Energy Intake (Kcal·d^− 1^)	Energy Balance (Kcal·d^− 1^)	Delta Body Mass (kg)
Combat Dive School	4567 (4332, 4803)	1748 (1672, 1824)	457 (433, 480)	2363 (2191, 2534)	2.61 (2.51, 2.72)	–	–	−0.39 (− 0.88, 0.10)
Pre-Mission Training	3904 (3589, 4219)	1798 (1738, 1859)	390 (359, 422)	1715 (1449, 1982)	2.17 (2.01, 2.34)	–	–	0.24 (−0.25, 0.73)
Weapons Training^a^	3682 (3076, 4289)	1868 (1756, 1979)	368 (308, 429)	1447 (949, 1944)	1.97 (1.68, 2.25)	3935 (3543, 4327)	253 (− 295, 801)	− 0.19 (− 1.29, 0.92)
Urban Combat^a^	5215 (4665, 5766)	1712 (1617, 1806)	522 (467, 577)	2982 (2528, 3437)	3.05 (2.74, 3.35)	2503 (2083, 2924)	− 2712 (− 3140, − 2284)	− 3.30 (− 4.77, − 1.81)
Squad Raids^a^	4801 (4426, 5175)	1838 (1732, 1943)	480 (443, 517)	2483 (2189, 2778)	2.62 (2.44, 2.80)	3118 (2619, 3616)	− 1683 (− 2281, − 1084)	−3.25 (− 4.62, − 1.88)
Platoon Raids^a^	4484 (3788, 5180)	1912 (1731, 2092)	448 (360, 535)	2554 (1731, 3377)	2.31 (2.07, 2.56)	4529 (4126, 4931)	45 (− 472, 561)	− 1.96 (− 2.63, − 1.29)
Small Unit Ranger Training (classroom)	3719 (3452, 3985)	1800 (1731, 1870)	372 (345, 399)	1546 (1349, 1744)	2.06 (1.95, 2.17)	3134 (2838, 3430)	− 584 (− 791, − 378)	–
Small Unit Ranger Training (field training)	4924 (4513, 5335)	1800 (1731, 1870)	493 (451, 534)	2631 (2303, 2959)	2.73 (2.55, 2.91)	2850 (2497, 3203	− 2074 (− 2491, − 1657)	–
Ranger Selection Assessment Program	4264 (4089, 4440)	1733 (1647, 1819)	426 (409, 444)	2105 (1999, 2211)	2.47 (2.39, 2.54)	2957 (2814, 3101)	− 1307 (− 1505, − 1110)	− 1.24 (− 1.69, − 0.78)
Raider Spirit^b^	6317 (5886, 6748)	1894 (1838, 1950)	632 (588, 675)	3791 (3416, 4167)	3.34 (3.11, 3.56)	2385 (2183, 2588)	− 3932 (− 4387, − 3477)	− 4.47 (− 5.40, − 3.54)
Close Quarters Battle^b^	4189 (3824, 4555)	1915 (1752, 2078)	419 (382, 455)	1856 (1636, 2076)	2.19 (2.07, 2.31)	2816 (2440, 3191)	− 1374 (− 1899, − 849)	−0.40 (− 1.79, 1.01)
Derna Bridge^b^	3736 (3546, 3926)	1936 (1870, 2002)	374 (355, 393)	1427 (1272, 1581)	1.93 (1.84, 2.03)	2701 (2256, 3146)	− 1035 (− 1481, − 589)	− 1.48 (− 2.27, − 0.70)
Overall	4468 (4311, 4624)	1827 (1801, 1853)	447 (431, 462)	2219 (2080, 2359)	2.45 (2.36, 2.53)	3086 (2941, 3231)	−1433 (− 1677, − 1188)	−1.75 (−2.09, − 1.40)

Data presented as mean (95% Confidence Interval of the mean)

^a^Data collected during US Army Special Forces Small Unit Tactics Training

^b^Data collected during Marine Special Operations Command

Resting Metabolic Rate = 370 + (21.6 * fat-free mass)

Diet-induced energy expenditure = 10% daily energy expenditure

Activity-Induced Energy Expenditure = Daily energy expenditure – (resting metabolic rate + diet-induced energy expenditure)

Physical Activity Level = Daily energy expenditure / resting metabolic rate

Energy Balance = Daily energy expenditure – energy intake

### Predicted energy expenditure

Regression analysis was conducted using the constant and β to create the below predictive equations.

$$ \mathrm{Model}\ \mathrm{A}\ \left(\mathrm{Kcal}\cdotp {\mathrm{d}}^{\hbox{-} 1}\right)=\left[47.97\ \mathrm{x}\ \mathrm{BM}\ \left(\mathrm{kg}\right)\right]+\left[706.33\ \mathrm{x}\ \mathrm{PAF}\right]\hbox{--} 467.22 $$

$$ \mathrm{Model}\ \mathrm{B}\ \left(\mathrm{Kcal}\cdotp {\mathrm{d}}^{\hbox{-} 1}\right)=\left[61.99\ \mathrm{x}\ \mathrm{FFM}\ \left(\mathrm{kg}\right)\right]+\left[716.49\ \mathrm{x}\ \mathrm{PAF}\right]\hbox{--} 721.30 $$

**Fig. 1:**
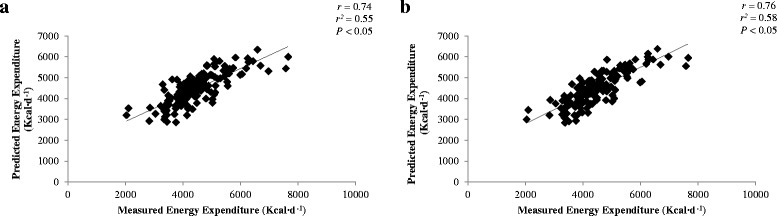
Correlation of energy expenditure as determined by doubly labeled water to predictive Model **a** and Model **b**

Where BM is body mass, PAF is physical activity factor, and FFM is fat-free mass. Model A (*r* = 0.74, *r*^*2*^ = 0.55) and Model B (*r* = 0.76, *r*^*2*^ = 0.58) were positively associated with measured daily energy expenditures (*P* < 0.05; Fig. 1) with the standard error of the estimate being 642 kcal·d^− 1^ of Model A and 626 kcal·d^− 1^ for Model B. Compared to average measured daily energy expenditure, predicted energy expenditure was not different for Model A [4463 (4337, 4588) Kcal·d^− 1^] or Model B [4462 (4333, 4591) Kcal·d^− 1^; Table 5]. However, compared to measured daily energy expenditure of the individual training course, Model A and Model B underestimated (*P* < 0.05) energy expenditure by 538 (88, 988) Kcal·d^− 1^ and 669 (219, 1119) Kcal·d^− 1^, respectively, for Raider Spirit.

**Table 5:** Comparison of measured daily energy expenditure to predicted daily energy expenditure models

Training	Measured (Kcal·d^−1^)	Model A (Kcal·d^− 1^)	Model B (Kcal·d^− 1^)
Combat Dive School	4567 (4332, 4803)	4857 (4569, 5145)	4663 (4375, 4951)
Pre-Mission Training	3904 (3589, 4219)	4225 (3960, 4490)	4092 (3827, 4357)
Weapons Training^a^	3682 (3076, 4289)	3734 (3458, 4009)	3574 (3298, 3849)
Urban Combat^a^	5215 (4665, 5766)	5528 (5209, 5846)	5276 (4958, 5594)
Squad Raids^a^	4801 (4426, 5175)	4767 (4491, 5042)	4921 (4645, 5196)
Platoon Raids^a^	4484 (3788, 5180)	4118 (3830, 4406)	4381 (4093, 4669)
Small Unit Ranger Training (classroom)	3719 (3452, 3985)	3352 (3087, 3617)	3381 (3116, 3646)
Small Unit Ranger Training (field training)	4924 (4513, 5335)	4765 (4500, 5030)	4814 (4549, 5079)
Ranger Selection Assessment Program	4264 (4089, 4440)	4610 (4378, 4841)	4621 (4390, 4853)
Raider Spirit^b^	6317 (5886, 6748)	5680 (5415, 5945)*	5799 (5534, 6064)*
Close Quarters Battle^b^	4189 (3824, 4555)	4457 (4139, 4776)	4426 (4107, 4744)
Derna Bridge	3736 (3546, 3926)	3719 (3454, 3984)	3769 (3504, 4034)
Overall	4468 (4311, 4624)	4463 (4337, 4588)	4462 (4333, 4591)

Data presented as mean (95% Confidence Interval of the mean)

^a^Data collected during US Army Special Forces Small Unit Tactics Training

^b^Data collected during Marine Special Operations Command

*Different than measured energy expenditure; *P* < 0.05

## Discussion

The primary finding of this integrative data analysis was that physical activity level accounted for the majority of the variation between the 12 SOF training operations for both daily energy expenditure (*r* = 0.91), energy balance (*r* = − 0.83), and change in body mass (*r* = − 0.47). Predictive equations for energy expenditure, separating physical activity level into quartiles coupled with body mass (Model A) or FFM (Model B), were correlated (*r* = 0.74 and *r* = 0.76, respectively) to measured energy expenditures with a standard error of the estimate of < 650 kcal·d^− 1^ for both Models. The predictive energy expenditure equation generated in this analysis will allow for development of interventions and appropriate military doctrine to encourage increased energy intake when physical activity level is anticipated to be high in an attempt to minimize negative energy balances and their associated physical performance decrements.

Energy expenditure during SOF training is quite variable depending on training activity, but averages ~ 4500 Kcal·d^− 1^ (range: 3700 to 6300 Kcal·d^− 1^). Moreover, SOF Service members experienced an average energy balance of − 1400 Kcal·d^− 1^ (range: 250 to − 3900 Kcal·d^− 1^) during training operations, resulting in a daily energy deficit of 30% total energy needs and declines in body mass that averaged 1.75 kg (range: 0.24 to − 4.47 kg) over the course of the training operation (4–10 days). Physical activity level was the factor that accounted for the majority of the variance in energy expenditure amongst the factors examined. These observations are not unique, as high physical activity levels and periods of negative energy balances are common during strenuous military training operations [8, 9, 15, 23–28]. The failure for SOF Service members to adequately increase energy intake to match energy expenditure is likely multifactorial. Standard field feeding protocol is to provide Service members with 3 MREs (~ 3600 Kcal·d^− 1^) per day [29]. If every component of the MREs is consumed, with an average energy expenditure of ~ 4500 Kcal·d^− 1^, SOF Service members would still be in a negative energy balance of ~ 900 Kcal·d^− 1^. As energy is limited by food availability, it is logical that personnel participating in SOF training with higher physical activity demands, and thus energy expenditures, would be the populations with a more severe negative energy balance. Mission objectives may also curtail time available to eat. In the present investigation, Urban Combat and Raider Spirit, trainings that produced the highest measured energy expenditures, also had the lowest energy intakes. These findings suggest that even if additional food is provided, if feeding is not appropriately integrated as part of training operations, it is unlikely that energy balance will be achieved [24]. Chronic or persistent under-eating and negative energy balance will not only result in undesired reductions in body mass, but can also compromise physical performance, and thus potentially mission readiness [30]. Therefore, there is interest in knowing which training events are the most energy demanding and where energy imbalance is most pronounced.

The derivation of a prediction algorithm that can accurately estimate the energy demands of SOF operations, is an important advance for better aligning food and energy availability with mission energy demands. Using regression analysis modeling and capturing physical activity level using a factor-based input, we were able to generate two predictive equations, one using body mass and PAF (Model A) and the other using fat-free mass and PAF (Model B) that had acceptable predictive accuracy. The generation of models using either fat-free mass or total mass was done to see if one or the other appeared to increase prediction acceptability and total mass was desirable in that is a variable that is relatively easy for the end user to capture. Additionally, simplifying activity estimates from measured physical activity levels (daily energy expenditure / daily resting metabolic rate) into four PAF will likely also aid in the practical use of these equations. As data collection for these past investigations were centered around specific training events, such as weapons training, land navigation, squad raids, and ambushes, the four PAF encompass common military tasks relevant to SOF groups across all branches of the military. Despite simplifying physical activity level into four discrete PAFs, the derived equations were highly associated to measured energy expenditures with a standard error of the estimate being ~ 14% (< 650 kcal·d^− 1^) of the mean for both Models. Though predictive algorithms derived in the current study provide estimates that overall were not different than measured daily energy expenditures during SOF training operations, the standard error of the estimate may be large enough to result in meaningful declines in performance [31]. Additionally, it is important to note that while both algorithms provided reasonable accuracy for prediction of average daily energy expenditure over the training periods in the data set, the PAF doesn’t capture the time domain, which is an important limitation of our algorithm that should be considered by SOF Service members using these equations. Furthermore, the resting metabolic rate equation used to calculate physical activity level was estimated from participant’s fat-free mass. Not having measurements of resting metabolic rate using indirect calorimetry may be a limitation in our model development and have contributed to the underestimated energy expenditure of the model for higher intensity operation. Use of fat-free mass in estimates of resting metabolic rate may also limit the generalizability to Soldiers with similar body compositions to those included in the current data set. Additional data are needed that contain more day-to-day variability in time spent in these military activities and heterogeneous sample size to properly estimate the accuracy and acceptability of these prediction algorithms.

The nutrition standards for the military personnel, known as the military dietary reference intakes (MDRIs), recommend the average male Service member consume 3400 Kcal·d^− 1^ for moderate activity in order to match intake to daily energy expenditures [29]. In context of this recommendation, SOF Service members must consume on average 135% (range: 108 to 185%) more energy than the MDRI to match daily energy expenditures during training operations. The Basic Daily Food Allowance (BDFA) is the amount of money provided per Service member for each meal service. Our findings suggest that the current BDFA allotted to SOF for personnel feeding (BDFA × 1.25) may be modestly too low to support the energy demands of personnel engaged in these training courses, based on greater energy requirements when compared to non-SOF personnel [32]. Increased funding may allow greater flexibility in menu planning potentially improving food options to stimulate energy intake and minimize energy deficits [1, 33]. Lastly, understanding when energy expenditures are anticipated to be elevated during training operations should prove useful to leaders to instill good food intake discipline within their units so as to ensure combat readiness.

While both predictive equations provided reasonably accurate estimations of the measured energy expenditures, they are not without limitations. The current investigation included data collected from 12 training operations in US Army Soldiers and Marines. As US Navy Special Warfare Command (WARCOM) and Air Force Special Operations Command (AFSOC) Service members’ data were not included, the generalizability of these equations to all branches within the US Special Operations Command (USSOCOM) remains to be determined. That said, given the wide variation of energy expenditures and military tasks included in the current investigation, it is likely that many of the tasks included would fall within the scope of activities in these other services and/or their unique activities would fall within the range of activities studied. Additionally, while our predictive equations were highly correlated with measured energy expenditures, there was an under prediction of total daily energy expenditure in the training activity with the highest energy demand (Raider Spirit, ~ 6300 Kcal·d^− 1^). Future studies will be needed to understand how best to resolve this error of estimation.

## Conclusion

In conclusion, we provide predictive algorithms that include body mass/fat free mass and a physical activity factor that are capable of predicting total daily energy demands of personnel engaged in an array of SOF training missions with acceptable accuracy. Based on these data, SOF Service members have energy requirements that exceed 135% (range: 108 to 185%) of the MDRI, at least during SOF training operations. These predictive equations provide nutrition professionals a tool to predict SOF energy expenditure with reasonable accuracy and logisticians to better align food delivery with energy requirements. The outputs of said tool are also of value for designing appropriate feeding strategies to support the nutritional requirements of the Warfighter.

## Abbreviations

AFSOC: Air Force Special Operations Command
DLW: doubly labeled water
FFM: fat-free mass
FM: fat mass
ITC: Individual Training Course
MARSOC: Marine Corps Forces Special Operations Command
MDRI: military dietary reference intakes
MRE: meals ready-to-eat
PAF: physical activity factor
PAL: physical activity level
RASP: Ranger Assessment and Selection Program
RMR: resting metabolic rate
SOF: Special Operations Forces
SURT: Small Unit Ranger Tactics
TBW: total body water
USSOCOM: the US Special Operations Command
WARCOM: US Navy Special Warfare Command
